# Endometriosis-associated bone loss: re-evaluating the potential role of systemic inflammation and hypoestrogenic therapies

**DOI:** 10.3389/fgwh.2026.1895443

**Published:** 2026-08-19

**Authors:** Farzaneh Motafeghi, Marzieh Saei Ghare Naz, Fahimeh Ramezani Tehrani, Samira Behboudi-Gandevani

**Affiliations:** 1 Reproductive Endocrinology Research Center, Research Institute for Endocrine Molecular Biology, Research Institute for Endocrine Sciences, Shahid Beheshti University of Medical Sciences, Tehran, Iran; 2 Foundation for Research & Education Excellence, Vestavia Hills, AL, United States; 3 Faculty of Nursing and Health Sciences, Nord University, Bodø, Norway

**Keywords:** bone health, bone mineral density (BMD), endometriosis, hypoestrogenism, inflammation, osteoporosis

## Abstract

Endometriosis is frequently managed with hormonal suppression that can induce hypoestrogenism during a period when many patients are still accruing peak bone mass. This narrative review summarizes current clinical and mechanistic evidence on bone health in endometriosis and its treatments and discusses a preliminary conceptual framework for risk stratification and monitoring. This review discusses two hypothetical converging pathways: (1) an intrinsic pathway in which chronic systemic inflammation may shift bone remodeling toward resorption (via cytokine-mediated effects on osteoclastogenic and Wnt signaling pathways and oxidative stress), and (2) an iatrogenic pathway in which ovarian suppression, most notably with gonadotropin-releasing hormone agonists/antagonists and some progestin-based regimens, reduces estrogen exposure and can lead to measurable short-term bone mineral density loss. Available observational data generally do not show clinically meaningful bone mineral density deficits or excess fracture incidence in untreated endometriosis, but treatment-associated bone mineral density loss is well documented; long-term fracture outcomes and bone-quality measures remain insufficiently studied. We compare the skeletal safety signals across commonly used therapies, discuss the role and limitations of add-back therapy, and highlight when baseline evaluation and follow-up assessment (including consideration of trabecular bone score) may be warranted in higher-risk patients.

## Graphical Abstract

**Figure:**
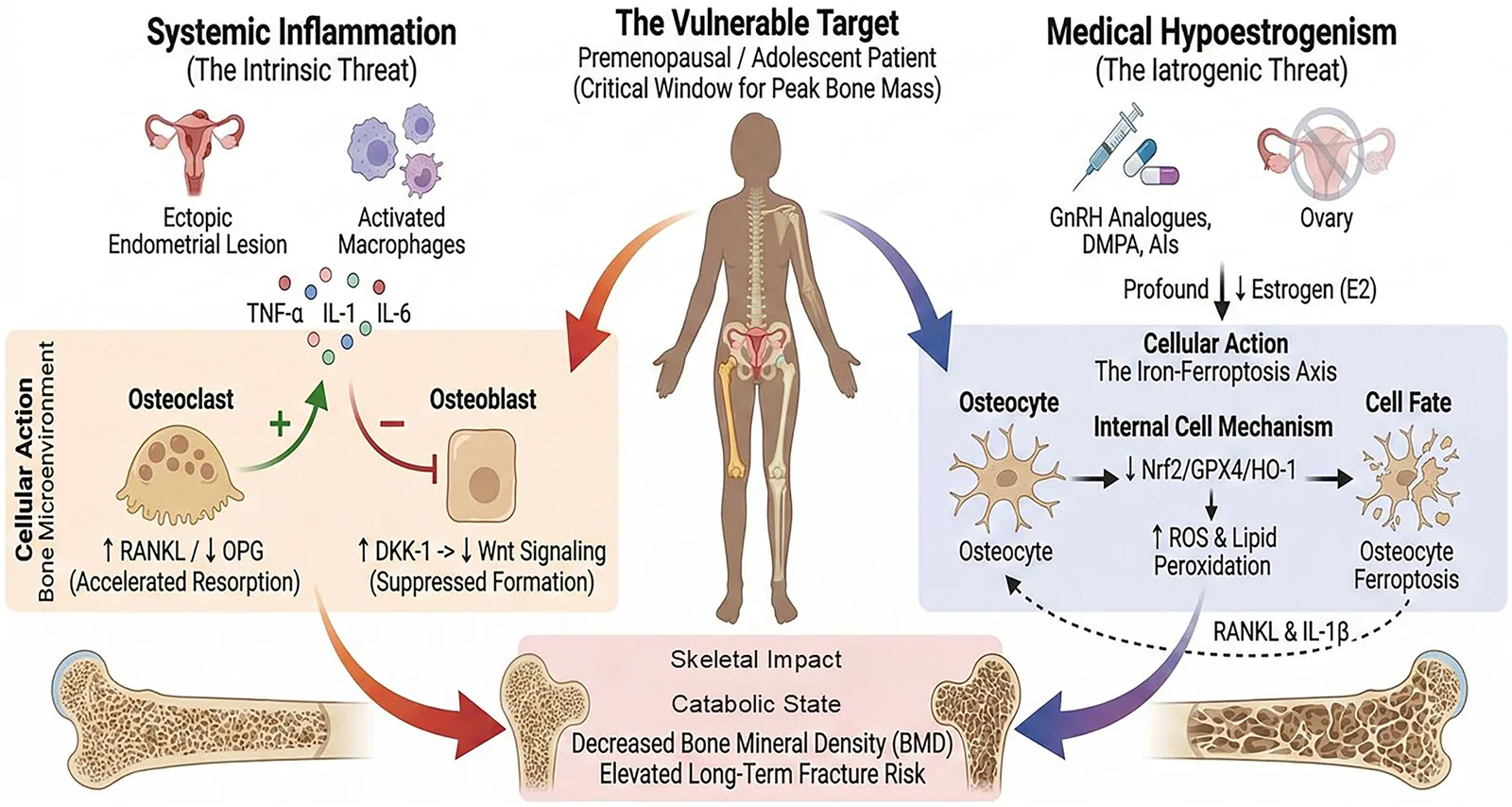
Proposed dual-pathway model linking endometriosis to impaired bone health. Endometriosis-associated systemic inflammation may influence bone remodeling through inflammatory, oxidative, and osteoclastogenic pathways, whereas hormonal therapies may cause treatment-induced hypoestrogenism and measurable bone mineral density loss. Individualized skeletal risk assessment and preventive strategies may help reduce treatment-related bone loss.

## 1 Introduction

Endometriosis is an inflammatory disease that affects an average of one in ten women of reproductive age (1–3). Treatment approaches are focused primarily on symptom relief, particularly chronic pelvic pain and infertility, as these problems significantly impact patients’ quality of life (4). This treatment approach tends to ignore long-term skeletal consequences, which may be due to underlying diseases or treatment effects. There appears to be a gap in current management, in which the effects of hypoestrogenism on bone health are rarely addressed in treatment plans for premenopausal women (5, 6).

This review considers the view that bone health may be influenced by two factors, the inflammatory environment caused by endometriosis and the consequences of its pharmacological treatments. Accordingly, this review advocates a comprehensive clinical approach that considers skeletal health as an essential part of the treatment plan. To this end, a two-factor conceptual model is proposed:

Endometriosis is associated with a systemic inflammatory environment characterized by increased levels of proinflammatory cytokines, including (Tumor Necrosis Factor-alpha) TNF-α, interleukin-1 (IL-1), and interleukin-6 (IL-6(. These cytokines may influence bone remodeling by increasing osteoclast-mediated bone resorption and reducing osteoblast-mediated bone formation (4, 7).

Some drug therapies, particularly gonadotropin-releasing hormone (GnRH) analogues, reduce ovarian estrogen production. This treatment-induced hypoestrogenic state is similar to the process of postmenopausal bone loss that occurs in young women and may coincide with the period of formation and attainment of peak bone mass (8, 9).

This review examines these two factors in detail. It first explains the pathophysiological mechanisms associated with the disease itself, then critically evaluates the skeletal effects of common hormonal therapies. It then presents the efficacy of novel therapies to maintain bone health. Finally, these findings are integrated to present an updated clinical algorithm that aims to prevent and promote skeletal health.

## 2 Endometriosis-Associated inflammation and bone remodeling

### 2.1 The clinical debate: is inherent bone loss truly prevalent

Before examining pathophysiological mechanisms, a clinical question must be answered: Does untreated endometriosis inherently increase the risk of osteopenia or osteoporosis?

Endometriosis-associated systemic inflammation has been proposed as a potential modifier of bone remodeling and, consequently, Bone Mineral Density (BMD) (4, 7).‏ However, this hypothesis is not consistently supported by the available clinical evidence.

A study by Lane et al. in 100 women with endometriosis, confirmed by laparoscopy, showed that the bone mineral density of the lumbar spine was within the normal range and was 103%–104% of the expected values in individuals of the same age. Therefore, there was no evidence of spontaneous bone loss (10). A controlled study by Dochi et al. also showed that, after adjusting for physical and lifestyle factors, there was no significant difference in bone density in any skeletal region between patients with endometriosis and healthy age-matched individuals (11). The long-term consequences of the disease were also examined in a large population-based study at the Mayo Clinic. Nearly 1,000 women were followed for 17 years. The results of this study showed that the cumulative incidence of fractures in this group (30.8%) was approximately the same as that expected in the general population (30.6%) and did not show a significant increase (12). Collectively, these findings suggest that endometriosis, in the absence of suppressive hormone therapy, does not necessarily lead to bone loss or an increased risk of fracture. However, the earlier BMD studies were relatively small and largely cross-sectional, limiting their ability to detect modest or progressive skeletal changes. The population-based fracture study provided longer follow-up and a larger sample, but its retrospective design, incomplete characterization of cumulative treatment exposure, and potential residual confounding should be considered when interpreting the absence of excess fracture risk.

### 2.2 Inflammation-Related bone changes in endometriosis

Although the link between endometriosis-induced inflammation and bone loss seems plausible from a biological perspective, clinical studies have not yet demonstrated a significant decrease in bone mineral density (BMD) or an increased risk of fracture in untreated patients. This discrepancy between biological evidence and clinical findings may have several reasons: first, the effect of inflammation on bone may be relatively modest at the population level and less evident in large studies; second, skeletal risk may be higher only in certain subgroups, such as patients with severe and inflammatory disease, low body mass index, adolescents, or those with comorbidities; third, dual-energy x-ray absorptiometry (DXA) bone density measurement may not be sensitive enough to detect micro-bone changes; and finally, many studies have not had sufficient follow-up to capture late outcomes such as fragility fractures (13). Therefore, these inflammatory pathways provide a more testable hypothesis for future longitudinal studies. In contrast, the most significant skeletal damage consistently reported to date is hypoestrogenism resulting from suppressive hormone therapies (4). Furthermore, the available evidence supports a clear distinction between mechanistic plausibility and clinically established skeletal risk (14). Inflammatory pathways may alter bone remodeling, but untreated endometriosis has not demonstrated lower BMD or increased fracture risk (10, 12). These mechanisms should therefore be viewed as hypotheses that require confirmation in well-designed longitudinal studies.

### 2.3 The inflammatory state

Endometriosis is increasingly recognized as a disease with systemic inflammatory features rather than a condition confined to the pelvis (4). The peritoneal fluid of affected patients contains activated macrophages and immune cells that secrete inflammatory cytokines, including TNF-α, IL-1, interleukin-6 (IL-6), and interleukin-17 (IL-17). If these mediators enter the systemic circulation, they may influence bone remodeling; however, the clinical significance of this pathway remains uncertain (15).

One potential pathway through which inflammation may influence bone remodeling is alteration of the receptor activator of nuclear factor kappa-B ligand (RANKL)/osteoprotegerin (OPG) axis (RANKL/OPG axis). RANKL is the main regulator of osteoclast differentiation and activation (14, 16). In contrast, OPG binds to RANKL, preventing it from binding to the receptor on osteoclast precursors. Thus, the balance between RANKL and OPG determines the final rate of bone resorption. Proinflammatory cytokines, particularly TNF-α and IL-1β, on the one hand increase RANKL expression in osteoblasts, osteocytes, and immune cells, and on the other hand decrease OPG production. The result of this dual effect is a disruption of the balance in favor of RANKL, increased osteoclast formation and activity, and ultimately increased bone resorption. Together, these effects may shift the RANKL/OPG balance toward greater osteoclast formation and bone resorption (17–20).

This inflammatory environment also suppresses bone formation. The Wnt signaling pathway is essential for normal osteoblast differentiation and function (21–25). TNF-α, one of the predominant cytokines in the inflammatory environment of endometriosis, stimulates the production of inhibitors of this pathway, in particular Dickkopf-1 (DKK-1). Increased DKK-1 inhibits Wnt pathway activity and reduces osteoblast maturation and function, leading to decreased bone formation (26, 27). Overall, these pathways provide a plausible mechanism through which endometriosis-associated inflammation may favor bone resorption over bone formation. However, whether these changes result in clinically meaningful bone loss or skeletal fragility in untreated patients remains uncertain, as studies have not consistently demonstrated reduced BMD or increased fracture risk (28, 29).

### 2.4 Shared genetic architecture: A predisposition to skeletal fragility?

Genetic studies suggest that endometriosis and bone-related or musculoskeletal and inflammatory traits may share some susceptibility loci and biological pathways (30, 31). However, the clinical relevance of these associations remains uncertain. Both susceptibility to endometriosis and baseline bone mineral density (BMD) are largely influenced by genetic factors, with approximately 50% of the risk of endometriosis (32, 33) and up to 80% of individual differences in bone mineral density heritable (34–37).

Genome-wide association studies have identified several loci and pathways that may be shared between endometriosis and skeletal or inflammatory traits. These findings suggest possible biological overlaps, but they do not establish a direct causal link with bone loss in patients with endometriosis (38, 39).

Several studies have identified genes such as BMPR2, BSN, and MLLT10 as common points of intersection between endometriosis and inflammatory bone diseases, including osteoarthritis and rheumatoid arthritis (30). In addition, the TNF-α, Wnt, and runt-related transcription factor 2 (RUNX2) -dependent transcriptional regulation pathways are involved in both immune response and bone remodeling (31, 40, 41). These data raise the possibility that some patients may have a dual vulnerability to both endometriosis-related inflammation and treatment-associated skeletal effects. However, this hypothesis has not yet been validated in clinical studies (42). In the future, genetic variants associated with BMD or inflammatory pathways may help inform treatment selection, although there is currently insufficient evidence to support their routine clinical use. If validated, genetic or biomarker-based risk profiles could eventually help identify patients who may require closer skeletal monitoring during long-term hypoestrogenic treatment. In such situations, local treatments such as the levonorgestrel-releasing intrauterine system (LNG-IUS) or the use of GnRH agonists with early and planned add-back therapy may be more appropriate (43).

Failure to integrate this genetic information into personalized treatment has resulted in the treatment approach for endometriosis remaining largely uniform and non-differentiated. While evidence supports the need for a risk-based approach, future studies should assess whether polygenic risk scores or biomarker panels can reliably predict treatment-related bone loss before they are considered for clinical decision-making.

## 3 Iatrogenic hypoestrogenism and skeletal risk in endometriosis therapies

Although endometriosis causes inflammation, the most important risk to bones is the severe reduction in estrogen caused by medication.

Doctors face a major dilemma in treating this disease: on the one hand, suppressing the hormone estrogen stops the activity of endometriosis and significantly reduces pain; on the other hand, estrogen deficiency accelerates bone tissue destruction and lowers bone density. Therefore, the choice of medication must be made with bone health in mind (44).

### 3.1 GnRH analogues and aromatase inhibitors

It is important to understand how estrogen depletion classically causes bone damage. Normally, the hormone estradiol (E2) protects bone. This effect is mediated largely through a receptor called estrogen receptor alpha (Er*α*). In osteoclasts, estrogen induces natural cell death, thereby reducing their lifespan and activity. In contrast, in osteoblasts and osteocytes, it prevents cell death so that these cells can build and maintain bone for longer (45).

When drugs such as GnRH analogues or aromatase inhibitors cause a sharp drop in estrogen, this balance is disrupted. This decrease in estrogen causes osteoclasts to live longer and destroy more bone, increasing RANKL expression and decreasing OPG levels. As a result, the RANKL/OPG ratio shifts towards greater osteoclast activation and accelerated bone destruction. Even without the inflammation of endometriosis, this hormonal decline alone can cause bone loss. Now, if chronic inflammation of endometriosis is added, the bone damage will be even more severe (46).

GnRH agonists such as Leuprolide acetate and antagonists such as Elagolix are among the most potent drug treatments for endometriosis. These drugs suppress the hypothalamic-pituitary-gonadal axis and put the body in a state similar to menopause, where estradiol levels drop dramatically. This dramatic drop in estrogen, while reducing endometriosis pain, is detrimental to bone. In just 6–12 months of treatment, GnRH agonists can cause a significant decrease in bone density in the lumbar spine, sometimes by as much as 6% (47). Aromatase inhibitors (AIs) also create a very low estrogen environment by preventing the conversion of androgens to estrogen. For this reason, these drugs are also associated with a rapid loss of bone density and an increased risk of osteoporotic fractures; the risk of fractures has been reported to be almost double that of natural menopause (48–50). For this reason, bone toxicity and damage from these drugs is considered an important clinical measure against which the bone risks of other hormonal therapies can be compared.

The evidence for short-term BMD loss with GnRH analogues is supported by clinical trials and comparative studies, although treatment regimens, add-back protocols, and follow-up periods differ across studies. Most reports assess BMD over 6–12 months and are not powered to evaluate fractures or long-term skeletal recovery. Evidence for aromatase inhibitors in endometriosis is more limited and is partly informed by studies in other estrogen-depleted populations, which may reduce its direct applicability to premenopausal women with endometriosis.

### 3.2 An emerging mechanistic hypothesis: the iron–ferroptosis axis

In addition to the well-known changes in bone formation and degradation, recent studies have suggested that the iron-ferroptosis axis may be one of the cellular links between severe estrogen depletion and bone damage. However, current evidence is mostly based on laboratory and preclinical studies and requires human studies. Ferroptosis is an iron-dependent form of cell death that is associated with severe oxidative damage to cell membranes (51).

Studies suggest that estrogen depletion may impair antioxidant pathways, including nuclear factor erythroid 2-related factor 2 (Nrf2), glutathione peroxidase 4 (GPX4), and heme oxygenase-1 (HO-1), and increase lipid peroxidation in bone cells. Experimental models suggest that ferroptotic or stressed osteocytes may release mediators such as RANKL and IL-1β, which could promote osteoclast activity. At present, there is no direct clinical evidence showing that ferroptosis contributes to bone loss in women receiving treatment for endometriosis (51, 52).

Existing studies suggest that estrogen depletion may reduce the activity of antioxidant pathways such as Nrf2, GPX4, and HO-1 in osteocytes and increase oxidative stress factors, especially lipid peroxidation factor, which increases the likelihood of ferroptosis in bone cells. Osteocytes that are stressed or undergo ferroptosis may produce or release more substances such as RANKL and IL-1β. These molecules stimulate osteoclasts and increase bone destruction. Therefore, the bone environment is altered towards increased bone resorption and resorption. Accordingly, it can be explained why the reduction of estrogen caused by hormonal treatments may weaken the antioxidant defense of bone cells and tip the balance of bone remodeling towards destruction. However, it is still unclear whether markers associated with ferroptosis can be identified in patients with endometriosis, and whether these changes are associated with decreased bone mineral density (BMD), changes in trabecular bone score (TBS), or increased fracture risk (51–53).

### 3.3 A critical deep dive on progestins

In the treatment of endometriosis, progestins are divided into two groups: DMPA is known to be a bone-damaging drug, while drugs such as DNG and NETA are often described as bone-neutral or low-risk (54–56). However, a close examination of the studies shows that this division is an oversimplification. In fact, each progestin has different hormonal and cellular properties and does not have the same effect on bone (57).

#### 3.3.1 Depot medroxyprogesterone acetate (DMPA)

Depot Medroxyprogesterone Acetate (DMPA) is one of the drugs with the most evidence of bone damage. This drug causes long-term estrogen depletion, reducing estradiol levels to approximately 26–35 pg/mL. As a result, bone mineral density (BMD) decreases in a duration-dependent manner (58, 59). Studies have shown that DMPA can cause a 6.6%–7.5% decrease in bone mineral density compared with controls, a rate that is approximately the same as that of GnRH agonists (60, 61). Because of these side effects, the U.S. Food and Drug Administration has issued a black box warning and recommends that DMPA not be used continuously for more than 2 years (62)^.‏^ The main debate about DMPA today is whether the bone loss is reversible after discontinuation of the drug. Some longitudinal studies have shown that some bone density may return to baseline within about 30 months after discontinuation of treatment (56, 57). However, other studies have shown that bone loss can persist for 2–5 years after stopping the drug, and in some patients, complete recovery does not occur, especially if use has been long-term (63). This is especially important in adolescents, because DMPA may be used during a period when the body is reaching peak bone mass. If bone loss occurs at this stage, the individual may never reach peak bone density and may be at increased risk of future fractures (64).

#### 3.3.2 Dienogest (DNG)

Dienogest, or DNG, is often touted as a safer alternative to DMPA, but scientific evidence suggests that it should be viewed with more caution. DNG is a relatively selective progestin and produces a modest reduction in estrogen, with estradiol levels typically remaining around 37 pg/mL (64). For this reason, in the past this level was still considered acceptable for bone health. However, several studies have reported significant decreases in lumbar bone mineral density, particularly in the first 6–12 months of treatment, with the rate of decrease reported to be 2.2%–2.7% (65).‏ Although this reduction is less than that with DMPA or GnRH analogues, it is still clinically important, especially in susceptible individuals such as adolescents. In a large study of adolescents, DNG use for 48 weeks resulted in a mean decrease of 1.2% in lumbar spine bone mineral density, with approximately 71% of participants showing bone loss compared to pretreatment (66). Although some bone mineral density is recovered 6 months after discontinuation of treatment, any impairment in bone formation during this growth period may be clinically significant (67, 68).‏ On the other hand, there is still insufficient information on the bone safety of long-term DNG use (>5 years). Some preliminary reports have suggested that the initial decrease in bone density may persist with continued treatment and may not change significantly after 60 months (69). However, these studies have important limitations, including the small number of patients, non-randomized design, and concomitant use of calcium and vitamin D supplements (70). Therefore, the current evidence is not yet sufficient to conclude that DNG is completely safe for bone in long-term treatment.

#### 3.3.3 Norethindrone acetate (NETA)

Norethindrone acetate (NETA) has a more complex pharmacological mechanism. In addition to its progestogenic effects, it also has some estrogenic and androgenic properties. This may be why it has a greater bone-protective effect. Part of the estrogenic effect of NETA is due to its partial conversion to ethinyl estradiol in the body, which can partially compensate for estrogen deficiency. Its androgenic properties may also have beneficial anabolic effects on bone (71, 72). Most studies of the bone benefits of NETA have been conducted as add-back therapy with GnRH agonists. In these studies, 5 mg daily of NETA, either alone or in combination with low-dose estrogen, prevented bone mineral density loss over 12 months and sometimes even increased BMD (73). However, this protective effect is not always definitive. In a comparative study, patients who received a GnRH agonist combined with NETA/estradiol experienced approximately the same rate of lumbar bone mineral density loss as patients who received DNG (−2.5% vs. −2.3%) (73, 74). This finding suggests that the protective effect of NETA is clinical-dependent and does not work equally well in all patients. Furthermore, long-term studies evaluating NETA as a primary treatment for endometriosis are lacking; therefore, its skeletal safety as standalone therapy remains uncertain.

Direct comparison of the skeletal effects of different progestins remains difficult because the available studies vary in design, population, treatment duration, comparator group, and use of calcium, vitamin D, or add-back therapy. Several studies are observational or include small samples, and most use change in BMD rather than fracture as the primary skeletal outcome. Differences between adolescent and adult populations also limit the generalizability of the findings. Accordingly, numerical changes in BMD across separate studies should not be interpreted as direct evidence that one progestin is definitively safer than another.

### 3.4 Unexplored avenues: direct anti-inflammatory osteprotection?

The main focus of studies on progestins has usually been on their hormonal effects and estrogen suppression. However, another important mechanism that has received less attention is the anti-inflammatory properties of progestins themselves. This is of great importance because chronic inflammation caused by endometriosis is a major factor in bone destruction. As previously mentioned, inflammatory cytokines such as TNF-α and IL-6 can increase osteoclast activity and enhance bone resorption. *In vitro* studies suggest that some progestins may reduce inflammatory signaling in endometriotic cells (75, 76).

In TNF-α-stimulated endometrial stromal cell culture models, DNG, NETA, and MPA significantly reduced the production of key inflammatory cytokines, such as IL-6 and IL-8, as well as MCP-1 (77). In addition, dienogest or DNG inhibited various inflammatory pathways and reduced the production of prostaglandins and cytokines, such as IL-1β (78). In experimental models, these effects appear to involve progesterone receptor signaling and reduced TNF-α-dependent responses (77).

These findings raise an important scientific question: Is it possible that the anti-inflammatory properties of some progestins may provide some bone protection, even though these drugs reduce estrogen? If this hypothesis is correct, it could explain why some drugs have less effect on bone than would be expected based on estrogen reduction alone. The comparatively modest BMD reductions reported in some dienogest studies raise the hypothesis that factors other than estrogen suppression may influence its skeletal effects. However, a contribution from anti-inflammatory activity has not been demonstrated clinically. It is possible that the anti-inflammatory effects of some progestins partly modify their overall skeletal impact. However, this possibility remains speculative and has not been demonstrated in clinical bone studies. Therefore, estrogen suppression may not be the only factor influencing the skeletal effects of progestins, although the contribution of anti-inflammatory activity remains uncertain (79).

In fact, the final outcome is likely to be the result of a complex balance between the intensity of the drug's hormonal suppression and its potential anti-inflammatory effects. However, there are still no robust clinical studies that can investigate these two mechanisms separately. This is a major gap in the current knowledge about the pharmacology of endometriosis and could be an important direction for future research and the design of less bone-threatening therapies (79).‏

Overall, the ferroptosis pathway, shared genetic susceptibility, pharmacogenomic risk prediction, and the potential anti-inflammatory skeletal effects of progestins should be considered emerging areas of research rather than clinically established mechanisms.

### 3.5 Comparative perspective on hormonal therapies

Combined hormonal contraceptives and the levonorgestrel-releasing intrauterine system (LNG-IUS) are commonly used for endometriosis-associated pain and are supported by current guidelines. Unlike GnRH analogues and aromatase inhibitors, these treatments generally do not cause marked systemic estrogen depletion. Combined hormonal contraceptives provide exogenous estrogen, whereas the LNG-IUS acts mainly through local progestogenic effects. Available evidence does not show consistent clinically important BMD loss in healthy adult users, although data on long-term fracture risk and bone effects in adolescents remain limited (43, 54, 64).

GnRH agonists and antagonists are effective for pain but produce greater estrogen suppression and are more consistently associated with BMD loss. Add-back therapy reduces this effect and is recommended when GnRH agonists are used. Recovery after treatment discontinuation varies, and complete return to baseline BMD cannot be assumed after prolonged treatment (43, 47, 80, 81).

Aromatase inhibitors also markedly reduce estrogen exposure. Although they may improve pain in patients with treatment-resistant endometriosis, the evidence is based mainly on small and short-term studies. Their long-term skeletal safety and the reversibility of BMD loss after discontinuation remain unclear. Current guidelines therefore reserve them for patients whose pain has not responded to other medical or surgical treatments (43, 48–50).

Progestins differ in their skeletal effects. DMPA is associated with the clearest reduction in BMD, dienogest may cause a smaller but measurable decline, and NETA-containing add-back regimens appear relatively bone-sparing, although evidence for NETA as standalone long-term therapy remains limited. Overall, treatment selection should consider pain control, contraceptive needs, degree of estrogen suppression, baseline skeletal risk, and expected treatment duration.

## 4 Challenging current mitigation strategies

Since the bone loss caused by hormonal therapy, especially GnRH analogues, is well known, the use of protective strategies has become an important part of the long-term treatment of endometriosis. The most important method used is add-back therapy, i.e., the simultaneous administration of low doses of steroid hormones to reduce the complications of severe estrogen deficiency. However, the theoretical basis of this method, as well as the evidence regarding its safety and long-term efficacy, still requires careful critical examination.

### 4.1 The estrogen window hypothesis for Add-back therapy

The main basis of add-back therapy is the estrogen window or estrogen threshold hypothesis, first proposed by Antonio R. Barbieri (82). According to this hypothesis, there is an ideal range for estradiol levels; about 30–50 pg/mL. In this range, estrogen is high enough to prevent menopausal symptoms and severe bone loss, but still low enough to inhibit the growth of endometrial lesions (82, 83).

Although this hypothesis makes sense in theory, its clinical implementation is much more complicated because not all add-back regimens are equally effective. For example, the route of estrogen administration is important. Oral estrogen can alter hepatic protein metabolism as it passes through the liver and reduce levels of IGF-1, a molecule that is important for bone health. In contrast, transdermal estrogen maintains hormone levels more consistently, has less impact on the liver, and does not increase the risk of venous thromboembolism (VTE) compared to the oral form. For this reason, the transdermal form is theoretically a safer and more appropriate choice for long-term bone protection. The type of progestin used with estrogen is also important. For example, norethindrone acetate (NETA) may help preserve bone matrix in addition to protecting the endometrium (84–86).

Short-term studies have shown that add-back therapy can significantly reduce the bone mineral density loss caused by GnRH agonists (87). Meta-analyses have also shown that this therapy significantly protects lumbar bone mineral density compared with treatment without add-back (88–90).

Despite its widespread use, there is still no robust, long-term evidence to define the estrogen window. Multi-year randomized studies that can demonstrate the true durability of this bone protection are very limited. For example, in a 24-month study of Elagolix with add-back therapy, the reduction in lumbar bone mineral density was only about −0.85%, which is a promising result (91). But even this amount still means bone loss. As a result, important questions remain unanswered: is this bone protection sustained over 5, 10, or 15 years of treatment? Is the “oestrogen window” the same in all patients? Or can factors such as age, genetic background, and type of add-back regimen alter it? On the other hand, there is another important concern that add-back therapy may inadvertently stimulate residual endometriosis lesions (43, 92, 93). This concern has led some physicians to prescribe very low doses of estrogen, doses that may not be sufficient to protect bone. However, strong scientific evidence to support this concern is still limited and based largely on anecdotal reports (94). Indeed, endometriosis itself has a high recurrence rate, even after surgery and suppressive therapy, reaching 20%–40% within 5 years (95). Therefore, it is not yet clear whether add-back therapy actually increases this baseline risk. Some studies have shown that 6 months of GnRH agonist treatment after surgery can reduce the risk of relapse (43, 81). However, it is not yet clear how the addition of add-back therapy affects this outcome. Overall, current decisions about add-back therapy are based more on theoretical concerns than on long-term, definitive evidence about bone safety and the risk of relapses.

Interpretation of the add-back literature is limited by variation in the type, dose, and route of the hormones used, as well as differences in treatment duration and baseline skeletal risk. Most trials were relatively short and focused on changes in lumbar spine BMD or hypoestrogenic symptoms rather than fracture outcomes. Attrition during longer follow-up and the limited number of direct comparisons between add-back regimens also make it difficult to identify an optimal strategy.

### 4.2 The missing endpoint: long-term skeletal outcomes after treatment cessation

The most important indicator of bone damage is not the decrease in bone mineral density (BMD) but the increase in the risk of fragility fractures. However, in many studies, there is a discrepancy between changes in BMD and fracture risk. This discrepancy has created a kind of paradox of fracture risk that calls into question some common assumptions in clinical management.

#### 4.2.1 The trajectory of BMD recovery

It is commonly assumed that bone loss caused by hormonal therapy is completely reversible. However, this assumption is not always true and may be overly optimistic. Long-term studies of GnRH analogs have shown alarming results. Results from a randomized clinical trial with a 6-year follow-up showed that there was a significant decrease in BMD during long-term treatment with a GnRH agonist. Despite discontinuation of treatment for 6 years, the decrease in BMD was not fully reversed, whether or not the patient received add-back therapy.‏ The results of these studies contradict the hypothesis that bone loss is completely reversible. In fact, even a long course of treatment may permanently alter the baseline level of bone health and create a permanent structural defect (96). A similar situation is seen with Depot Medroxyprogesterone Acetate, or DMPA. Although many patients have some degree of bone remodeling after stopping the drug, the results of studies are not entirely consistent. Some groups of patients continue to have decreased bone density many years after stopping treatment, especially those who have been taking the drug for a longer period of time (63). Furthermore, the process of bone regeneration itself is slow and time-consuming, and may take several years. During this time, the individual remains in a vulnerable bone state and the risk of fracture can remain elevated (97, 98).

#### 4.2.2 The fracture risk paradox

While there is some evidence that hormone therapy can cause bone loss, recent studies have suggested a completely different conclusion. In a large population-based study from the Mayo Clinic, nearly 1,000 women with surgically confirmed endometriosis were followed for more than 17 years. The results showed that the overall risk of bone fracture was not increased over the long term in this population. In fact, the cumulative fracture rate over 20 years was 30.8%, which is about the same as the expected rate in the general population (30.6%).‏ If we accept this finding without critical interpretation, it might be thought that concerns about treatment-induced bone loss are not clinically significant. However, this result raises an important “paradox”: how is it possible to see a decrease in BMD but not a significant increase in fractures? Several hypotheses have been proposed to explain this discrepancy. First, the observational design of this study could leave confounding factors undetected. This means that factors other than the drugs themselves may have influenced fracture risk (12). For example, independent studies of users of Depot Medroxyprogesterone Acetate, or DMPA, have shown that these individuals may have an inherently higher risk of fracture even before treatment begins. This suggests that factors such as socioeconomic status, lifestyle, nutrition, physical activity, or other underlying characteristics may contribute to the results, not just the medication (99). Although the Mayo Clinic study accounted for factors such as corticosteroid use in its statistical analysis, there is still the possibility that hidden, uncontrolled factors could have influenced the results (12).

A second explanation for this paradox is that the study follow-up period may not have been long enough to observe the fracture outcome. In the Mayo Clinic study, the mean age of diagnosis was about 35 years, and the 20-year follow-up followed them only until about 55 years of age. However, the consequence of reduced peak bone mass is usually apparent at an older age, especially after 65 years, when age-related bone loss and menopause are at their peak. Therefore, the study follow-up period may have ended before the late effects of treatment-induced bone damage had a chance to manifest (100, 101).

#### 4.2.3 Bone microarchitecture and bone quality beyond BMD

Areal BMD measured by DXA remains the main clinical measure of bone mass, but it does not capture all factors that determine bone strength. Bone strength also depends on trabecular number, thickness, separation, and connectivity, as well as cortical thickness, density, and porosity. Changes in these features may occur without a marked reduction in BMD and may partly explain why changes in BMD do not always correspond to fracture outcomes (102, 103).

Cortical bone quality is particularly relevant because thinning of the cortex and increased cortical porosity can reduce bone strength. However, conventional DXA cannot separate cortical from trabecular bone or directly assess these structural changes. High-resolution peripheral quantitative computed tomography (HR-pQCT) provides three-dimensional measures of volumetric BMD and bone microarchitecture at peripheral sites, mainly the distal radius and tibia. It can assess trabecular number, thickness, and separation, as well as cortical thickness and porosity. Bone strength can also be estimated from HR-pQCT images using finite element analysis (103).

Studies in older populations have shown that HR-pQCT measures of cortical and trabecular microarchitecture may provide information on fracture risk beyond areal BMD. However, evidence in premenopausal women and patients receiving treatment for endometriosis is limited. HR-pQCT is also not widely available, and its use is mainly restricted to research settings. Longitudinal studies are needed to determine whether endometriosis or estrogen-suppressive treatments affect cortical and trabecular bones differently and whether these changes recover after treatment is stopped (103, 104).

Trabecular bone scores are derived from the gray-level texture of lumbar spine DXA images and provide indirect information related to trabecular bone structure. It may add information to BMD-based fracture assessment, but it is not a direct measure of trabecular microarchitecture. Its role in young and premenopausal women is less clear, and it has not been validated for monitoring hormonal treatment in endometriosis. Therefore, TBS should currently be considered a possible research or supplementary measure rather than a routine monitoring tool in this population (80, 105, 106).

Future studies should combine DXA with measures of bone quality, such as TBS and HR-pQCT, and include both cortical and trabecular outcomes. These studies should also assess treatment duration, recovery after treatment cessation, bone turnover markers, and long-term fracture outcomes. Such an approach may help determine whether treatment-related reductions in BMD reflect temporary changes in bone mass or more persistent changes in bone structure.

Long-term studies that follow patients into the postmenopausal years and include fragility fractures as a primary outcome are still needed (102).

### 4.3 Quality and limitations of the available evidence

The available evidence has several important limitations. Most studies on bone health in untreated endometriosis are observational, and many include small samples. Some studies also do not fully account for factors that can affect bone health, such as age, body mass index (BMI), physical activity, nutrition, smoking, and previous hormonal treatment. Population-based studies usually include more participants and longer follow-up, but retrospective studies may still be affected by confounding, incomplete treatment data, and differences in how fractures are identified.

Studies on treatment-related bone loss also vary widely. They differ in patient age, treatment type, dose, duration, skeletal site assessed, use of add-back therapy or supplements, and timing of follow-up DXA. Many trials were mainly designed to assess pain relief or treatment response, with bone outcomes included only as secondary measures. Most studies focus on short-term changes in BMD, while fracture risk, bone microarchitecture, and bone recovery after treatment are less often studied. Small sample sizes, non-randomized designs, loss to follow-up, and the lack of suitable comparison groups also make it difficult to compare treatments directly.

These limitations do not question the observed reduction in BMD with strong estrogen suppression. However, they make it difficult to determine the exact size of this effect, whether bone loss is fully reversible, and how it affects long-term fracture risk. Therefore, the clinical suggestions in this review should be viewed as a risk-based approach rather than fixed recommendations for all patients.

## 5 A New clinical framework: integrating bone health stewardship into endometriosis care

The current evidence on bone health in patients with endometriosis still faces important limitations. Inconsistency between study results, lack of long-term data, and overreliance on indirect indicators such as BMD make it difficult to judge with complete confidence the bone safety of endometriosis treatments throughout life. On the other hand, if we wait until multi-decade studies on fracture rates are published, the opportunity for early intervention and effective prevention of bone damage may be missed. For this reason, there is a growing need to shift from the current approach of reactive management of injury to preventive, risk-based bone preservation. This shift in approach means that bone health care should not be an afterthought in endometriosis treatment but should become a consistent and essential part of routine patient care. Accordingly, the proposed algorithm that follows is designed as a practical, evidence-based tool to achieve this goal in the clinical management of patients.

Given the methodological limitations and limited long-term outcome data, the following framework should be interpreted as a proposed risk-based approach rather than a formally validated clinical guideline.

### 5.1 Proposed conceptual framework for future clinical validation

The following framework combines current guideline recommendations with the authors’ proposed approach. Guideline-supported measures include assessment of clinical risk factors and the use of add-back therapy with GnRH agonists. In contrast, the use of TBS, bone turnover markers (BTMs), and specific DXA monitoring intervals has not been established in endometriosis guidelines. These measures are therefore presented as possible options for selected high-risk patients and require further clinical evaluation (43).

Step 1: Suggested Baseline Assessment
  • Before starting treatment, clinical risk factors for bone loss should be reviewed, particularly when the planned therapy causes marked or prolonged estrogen suppression (107). This approach moves bone health from a secondary issue to a core part of the initial evaluation of patients with endometriosis.
  • **Clinical risk factor assessment:** The physician should systematically assess all factors that may increase the risk of osteoporosis independently of endometriosis. These factors include a family history of osteoporosis or fragility fractures, low BMI (BMI < 18.5 kg/m²), history or presence of eating disorders, smoking, excessive alcohol consumption, and past or current use of bone-damaging medications, especially glucocorticoids (107).
  • **Baseline DXA (selected patients):** Baseline DXA is not routinely required for all patients. It may be considered in patients with a history of low-trauma fracture, known low BMD, major clinical risk factors, or planned prolonged or repeated treatment with a strongly estrogen-suppressive therapy. When DXA is performed in premenopausal women, Z-scores should be used for interpretation (108).
Step 2: Skeletally Informed Therapy Selection

The choice of hormonal therapy should be a shared, individualized decision. In this process, the patient's baseline risk of bone loss needs to be considered alongside the effectiveness of the treatment and the potential side effects of each drug.

- **Lower skeletal risk:** In patients who do not have significant risk factors for osteoporosis and whose baseline bone density is normal (if DXA is performed), the range of treatment options is broader. In this group, the choice of drug is based on the severity of endometriosis symptoms, patient preference, and need for contraception.‏
- **Higher skeletal risk:** In patients with a pre-existing bone lesion, more careful treatment selection is necessary. For example, an adolescent with a strong family history of osteoporosis and a low Z-score may be at higher risk for bone loss. In such situations, the priority of treatment may be more focused on bone preservation.
  ○ **First-line options** In these patients, treatments that provide minimal systemic estrogen suppression may be a more appropriate choice, such as the levonorgestrel-releasing intrauterine system (LNG-IUS), which has a predominantly local progestational effect and has a more limited systemic hormonal effect (109).
  ○ **Second-line option:** The next option could be systemic progestins with a milder bone profile, such as norethindrone acetate, dienogest, although in these situations it is better to define a specific program for periodic monitoring of bone density (DXA monitoring) from the beginning (54, 110, 111).
  ○ **High-risk interventions:** Treatments such as Depot Medroxyprogesterone Acetate (DMPA) and long-term GnRH agonist therapy without add-back therapy may be better considered as alternative or next-line options in high-risk patients, rather than the first choice (96, 112).

Step 3: Therapy-Specific BMD Monitoring

Current endometriosis guidelines do not define standard bone-monitoring intervals for individual hormonal treatments. The following considerations represent a risk-based approach proposed by the authors.

- Bone turnover markers: C-terminal telopeptide of type I collagen (CTX) and procollagen type I N-terminal propeptide (P1NP) are commonly used markers of bone resorption and formation, respectively. However, their role in monitoring bone health during endometriosis treatment has not been established. Routine measurement of these markers cannot currently be recommended, but they may be useful in research or selected clinical cases (113, 114).
- **GnRH Agonists/Antagonists:** These treatments may reduce BMD, particularly with longer exposure. Follow-up DXA may be considered in patients with major skeletal risk factors or in those receiving prolonged or repeated treatment. A standard monitoring interval has not been established (43, 47, 96).
- **Depot Medroxyprogesterone Acetate (DMPA):** DMPA use is associated with a reduction in BMD. However, routine DXA monitoring is not recommended solely because of DMPA use. Individual assessment may be considered when other major risk factors for bone loss are present (55)
- **Dienogest (DNG):** A modest reduction in lumbar spine BMD has been reported during dienogest treatment, particularly during the first year. No standard DXA monitoring interval has been established. Follow-up assessment may be considered in adolescents, patients with additional skeletal risk factors, or those receiving prolonged treatment (65–67).
- **Combined hormonal contraceptives:** Routine DXA monitoring is generally not required in patients without additional skeletal risk factors (54, 73, 110).
- **Norethindrone acetate:** Available add-back studies suggest a relatively favorable skeletal profile, but long-term data for NETA as primary endometriosis therapy remain limited (54, 73, 110).
- **Aromatase Inhibitors (AIs):** Because these treatments markedly reduce estrogen exposure, baseline and follow-up BMD assessment may be considered, particularly in patients with additional skeletal risk factors. However, no specific DXA monitoring interval has been established for their use in endometriosis (49, 50, 115).

Step 4: Proactive Mitigation and Lifestyle Counseling

Skeletal preservation strategies would ideally operate preemptively, functioning as a potential framework for care rather than a reactionary measure deployed only post-injury.

**Figure 1:**
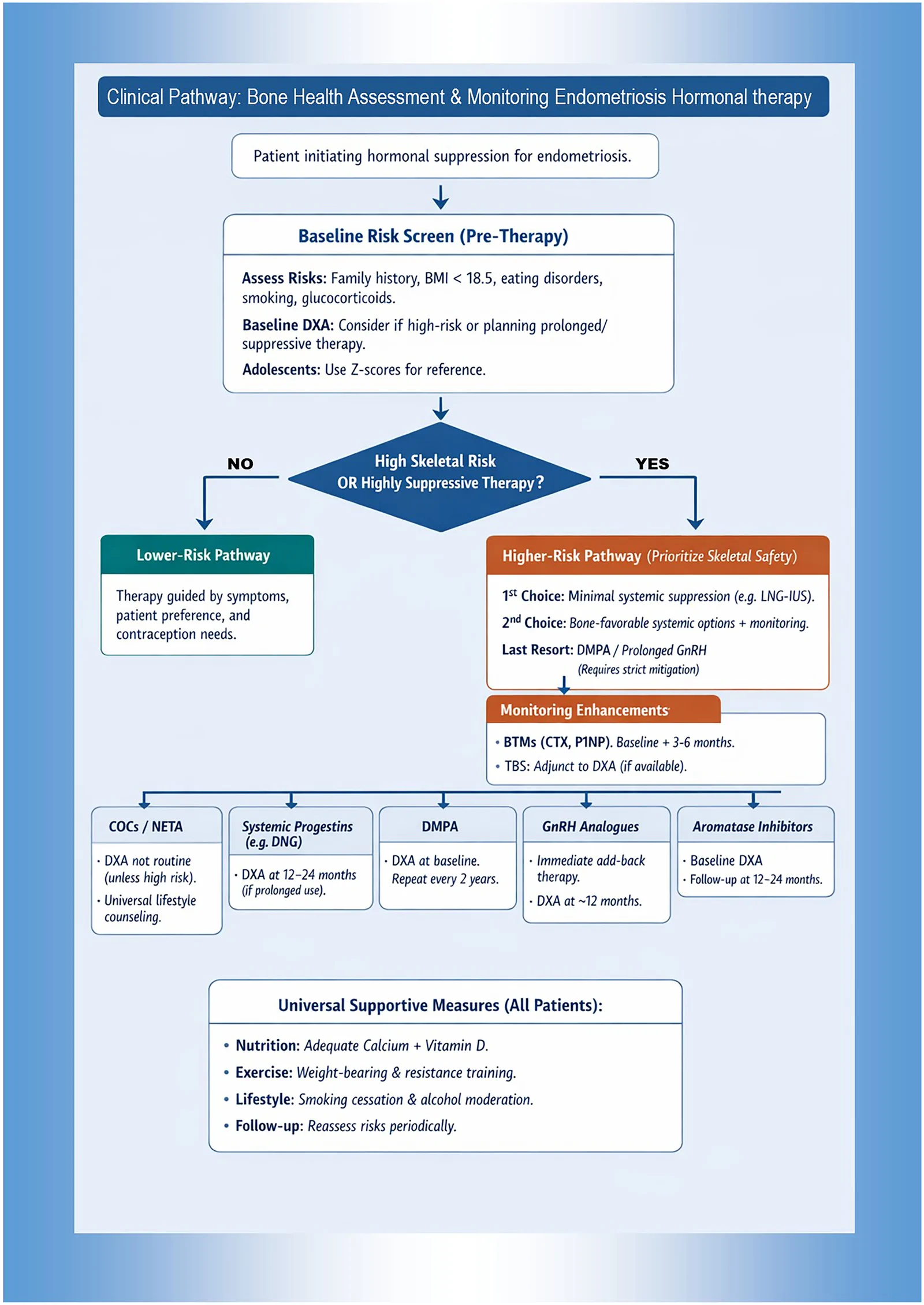
Proposed risk-based framework for integrating bone health assessment into endometriosis management. The framework includes baseline skeletal risk assessment, individualized treatment selection, therapy-specific monitoring, and preventive measures such as add-back therapy and lifestyle counseling. The proposed approach is conceptual and requires prospective clinical validation.

- **Immediate Add-Back Therapy:** For individuals commencing GnRH agonist regimens, the concurrent initiation of add-back supplementation is conceptually highly recommended alongside the primary pharmacological agent. Delaying the introduction of add-back therapy exposes the patient to an initial, rapid phase of bone demineralization that may ultimately prove structurally irreversible (116, 117).
- **Universal Lifestyle Counseling:** Irrespective of the specific therapeutic trajectory, all patients navigating an endometriosis diagnosis would likely benefit from comprehensive education regarding bone-protective lifestyle behaviors. This encompasses verifying adequate nutritional or supplemental assimilation of calcium (1,000–1,300 mg/day) and vitamin D (600–800 IU/day), maintaining a consistent regimen of weight-bearing and resistance-training exercises, enacting smoking cessation, and stringently moderating alcohol consumption (118). Specifically for individuals concluding GnRH agonist therapy, targeted physical training has demonstrated notable efficacy in accelerating osteological remineralization and should be strongly encouraged by attending clinicians (119) (Figure 1).

This framework does not represent an inflexible clinical protocol, but rather a conceptual realignment. It deliberately re-centers the enduring, systemic well-being of the patient, functioning on the premise that the competent management of a chronic inflammatory pathology intrinsically requires the proactive management of its unintended iatrogenic consequences. Ultimately, it elevates skeletal integrity from a mere clinical footnote to a central pillar of endometriosis management.

## 6 Conclusion

The relationship between endometriosis and skeletal health appears to involve two potentially converging pathways: disease-associated systemic inflammation and treatment-induced hypoestrogenism. Although inflammatory cytokines may theoretically promote bone resorption and suppress bone formation through the RANKL/OPG and Wnt signaling pathways, current clinical evidence does not consistently demonstrate reduced bone mineral density or increased fracture risk in untreated women with endometriosis. Therefore, the independent skeletal contribution of endometriosis-associated inflammation remains uncertain.

In contrast, bone loss associated with estrogen-suppressive therapies is more consistently documented. GnRH agonists and antagonists, aromatase inhibitors, and depot medroxyprogesterone acetate may produce clinically relevant reductions in bone mineral density, particularly with prolonged exposure and in adolescents or patients with pre-existing skeletal risk factors. Dienogest may also cause modest bone loss, whereas evidence regarding the long-term skeletal safety of other progestin-based regimens remains limited. Add-back therapy can attenuate treatment-related bone loss, but its long-term effects on fracture risk and bone recovery after treatment cessation are insufficiently established.

Bone health assessment in endometriosis should therefore be individualized according to age, baseline risk factors, treatment type, and expected duration of therapy. Prospective studies incorporating fracture outcomes, bone microarchitecture, treatment exposure, and post-treatment recovery are required to clarify the long-term skeletal consequences of endometriosis management and to validate risk-based monitoring strategies.

## Data Availability

All data supporting the findings of this review published before and are available within the paper and its reference list.
